# Analysis of the Structural Organization and Expression of the *Vrn-D1* Gene Controlling Growth Habit (Spring vs. Winter) in *Aegilops tauschii* Coss.

**DOI:** 10.3390/plants12203596

**Published:** 2023-10-17

**Authors:** Grigory Yurievich Chepurnov, Ekaterina Sergeevna Ovchinnikova, Alexander Genadevich Blinov, Nadezhda Nikolaevna Chikida, Mariya Khasbulatovna Belousova, Nikolay Petrovich Goncharov

**Affiliations:** 1 Early Maturity Genetics Laboratory, Institute of Cytology and Genetics, Siberian Branch of the Russian Academy of Sciences, Akademika Lavrentieva Avenue, 10, 630090 Novosibirsk, Russia; ovchinnikova@bionet.nsc.ru (E.S.O.); blinov@bionet.nsc.ru (A.G.B.); 2 Division of Wheat Genetic Resources, Federal Research Center N. I. Vavilov All-Russian Institute of Plant Genetic Resources (VIR), 190000 Saint Petersburg, Russia; n.chikida@vir.nw.ru; 3 Wheat Laboratory, Dagestan Experimental Station—The Branch of the Federal Research Center N. I. Vavilov All-Russian Institute of Plant Genetic Resources, Vavilovo Village, Derbent District, 368600 Saint Petersburg, Russia; m.h.belousova@mail.ru

**Keywords:** *Aegilops tauschii*, *Vrn-D1*, *ZCCT-D1*, *ZCCT-D2*, *Ppd-D1*, expression level, growth habit

## Abstract

The duration of the vegetative period is an important agronomic characteristic of cereal crops. It is mainly influenced by the *Vrn* (response to vernalization) and *Ppd* (response to photoperiod) genes. In this work, we searched for alleles of several known genes of these two systems of response to external conditions in 15 accessions of *Aegilops tauschii* Coss. (syn. *Ae. squarrosa* L.), with the aim of studying the impact these alleles have on the vegetative period duration and growth habit. As a result, three allelic variants have been found for the *Vrn-D1* gene: (i) one intact (winter type), (ii) one with a 5437 bp deletion in the first intron and (iii) one previously undescribed allele with a 3273 bp deletion in the first intron. It has been shown that the spring growth habit of *Ae. tauschii* can be developed due to the presence of a new allele of the *Vrn-D1* gene. Significant differences in expression levels between the new allelic variant of the *Vrn-D1* gene and the intact allele *vrn-D1* were confirmed by qPCR. The new allele can be introgressed into common wheat to enhance the biodiversity of the spring growth habit and vegetative period duration of plants.

## 1. Introduction

The duration of the vegetative period is one of the most important adaptive and agronomic traits of cereal crops, which has a substantial impact on plant productivity and minimizes the impact of biotic and abiotic stresses [1]. Cereal crops have three signal systems of response to external conditions that affect the duration of plants’ vegetative period (earliness): (i) response to vernalization (*Vrn* genes), (ii) response to photoperiod (day length) (*Ppd* genes) and (iii) earliness per se (*Eps* genes) [2,3,4].

**Figure 1:**
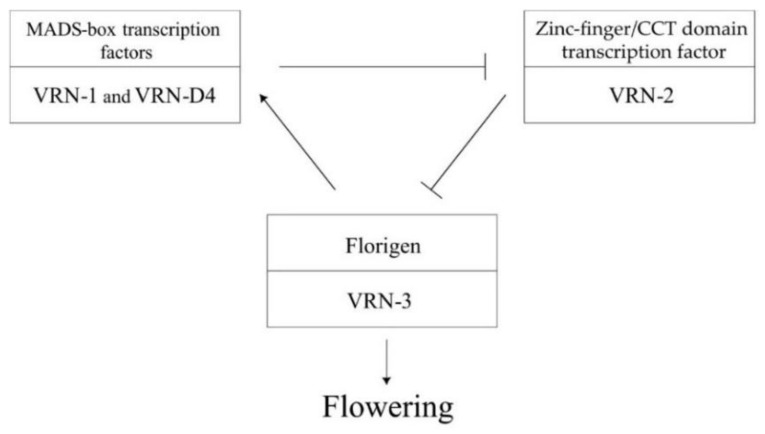
Diagram of interaction between *VRN* loci in leaves. The *VRN-1* and *VRN-D4* loci suppress the *VRN-2* locus, which leads to the appearance of florigen (*VRN-3* locus). The *VRN-3* genes’ protein is then transported through the phloem to the shoot apical meristem where it induces the meristem-identity genes and the initiation of the reproductive stage.

Vernalization is an impact of low positive temperatures, which is necessary to initiate spike formation [5]. This is necessary to protect the floral meristems of winter plants from damage by negative temperatures in winter. Spring plants (S), which do not require exposure to low temperatures to initiate flowering, turned out to be more suitable for cultivation in the continental regions of the northern and southern latitudes from 40° to 60° [6]. Genetic control of the response to vernalization in *Ae. tauschii* has been poorly studied. Most studies of the genetic regulation of these systems of response to external conditions have been carried out in the hexaploid wheat *Triticum aestivum* L., and so most of the current studies in this area are based on the loci identified in this species. In common wheat, four *Vrn* gene loci have been identified (*VRN-1*, *VRN-2*, *VRN-3* and *VRN-D4*) and are noted for the following: changes to their structure cause differences in growth habit and response to vernalization [7,8,9,10,11,12,13]. The *VRN-1* genes (*Vrn-A1*, *Vrn-B1*, *Vrn-D1*) and their paralog *Vrn-D4* (the *VRN-D4* locus) are MADS-box transcription factors homologous to the *APETALA-1* (*AP-1*) gene in *Arabidopsis thaliana* (L.) Heynh. [7,13,14,15]. Expression of these genes in the leaves initiates the heading of wheat plants [16]; however, without exposure to low temperatures, their pre-mRNA binds to the RNA-binding protein TaGRP2, homologous to GRP7 in *Arabidopsis*, so the intact alleles are expressed at a low basal level [12,13,17]. When wheat plants grow at low positive temperatures, TaGRP2 modifies O-GlcNAcylation, allowing it to interact with a carbohydrate-binding protein called VER2. The interaction between VER2 and O-GlcNAc-TaGRP2 reduces the amount of TaGRP2 in the nucleus, leading to the accumulation of the mRNA of the *VRN-1* genes [17]. The processes described are typical of winter varieties; however, mutations in the first intron of the *VRN-1* and *VRN-D4* genes, which affect TaGRP2 binding sites, as well as mutations in the promoters of the *VRN-1* genes allow them to be expressed without exposure to low temperatures and thus to confer a spring growth habit [8,11,18,19,20,21,22,23,24,25,26]. The interaction diagram of *VRN* genes is shown in Figure 1. It has been shown that the main role of proteins encoded by the *VRN-1* genes in the leaves is decreasing the expression levels of the *Zinc-finger/CCT domain transcription factor-1* (*ZCCT-1*) and *Zinc-finger/CCT domain transcription factor-2* (*ZCCT-2*) genes (*VRN-2* locus) [16]. The *VRN-2* genes encode proteins containing zinc finger domains and CCT domains that suppress the expression of the *VRN-3* genes in wheat and barley [18,27]. The *VRN-3* locus contains genes homologous to the *FLOWERING LOCUS T* (*FT*) gene in *Arabidopsis thaliana* (named *TaFT* in wheat) known as florigene [10]. In wheat, these genes encode polypeptides that can bind to FDL2 proteins [28] and proteins in the 14-3-3 family [29] and are transported as a single complex from leaves to the apical meristem, where they interact with the promoter region of the *VRN-1* genes [7,14]. This causes the expression of the *VRN-1* genes in the growth cone, which leads to the transition from the vegetative to the generative phase of development [15,28]. It has been demonstrated that a spring growth habit can be conferred by mutations in the *VRN-1*, *VRN-D4* and *VRN-3* genes, which govern their expression without exposure to low positive temperatures (vernalization) [10,13,16], or due to mutations reducing the number of proteins encoded by the *VRN-2* genes or changing their amino acid composition [30,31,32].

Not only the *Vrn* genes but also the *Ppd* and *Eps* genes contribute to changes in the duration of the vegetative period. Of the *Ppd* genes, the ones that have the greatest influence on the trait are the homologous genes at the *PPD-1* locus [33,34,35,36,37,38], which belong to the pseudoresponse regulator (PRR) family [37,39,40,41,42]. Plants can develop earliness due to large deletions or insertions of transposons in the promoter region of the *PPD-1* genes [37,38,43,44] as well as due to changes in the copy number of these genes (CNVs) [35]. Earliness per se (*Eps*) is the difference in the heading time of the varieties that do not show differences in response to vernalization or photoperiod changes [45]. Earliness per se is controlled by a number of minor genes, the effects of which can be determined only in the absence of vernalization and response to photoperiod effects [46]. Although the genes and QTLs (quantitative trait loci) for earliness per se have been little studied [46], they are known to increase developmental rates in any growth phase [47].

It has been shown that the *Vrn* and *Ppd* genes are the main contributors to changes in the duration of the vegetative period of cereal crops [3,4,46]. Furthermore, they can shorten the vegetative period in the presence of the dominant alleles of the *Vrn* and *Ppd* genes in the plant genome, either alone [37,48,49] or in combination [48,49]. The genes at these loci found in *T. aestivum* L. have been poorly studied in the donor species of the elementary genomes of common wheat; however, they may contain valuable alleles of the *Vrn* and *Ppd* genes. Because information about their alleles is scarce, so is the pool of those from which plant breeding can benefit. *Ae. tauschii* is the donor of the D genome of hexaploid wheats [50,51,52,53,54,55,56,57]. Most accessions of this species represent plants with a winter growth habit [58,59,60]; however, some of the spring accessions that were found there, too, had rather short vegetative periods [58,59,60].

Only a few mutations in the structure of the *Vrn-D1* gene (*VRN-1* locus) and the *ZCCT*-*D1* and *ZCCT*-*D2* genes (*VRN-2* locus) of *Ae. tauschii* have been described [30,61,62]. Detection of mutant alleles of the *Vrn-D3* gene (*VRN-3* locus) has not yet been reported. The sequence of the *Vrn-D4* gene of *Ae. tauschii* is not yet known [13]. It has been shown that the *Vrn-D4* sequence is present only in the D genome of common wheat, where it developed due to a translocation between the long arm of chromosome 5A and a proximal region of 5DS [13]. As was shown in wheat, spring varieties contain mutant alleles of the *VRN-1* genes with various deletions and insertions in the promoter region and deletions in the first intron [8,11,18,19,20,21,22,23,24,25,26]. No changes in the promoter region of the *Vrn-D1* gene in spring *Ae. tauschii* accessions have been found [26]. At the same time, two mutations in the first intron have been described: a 5437 bp deletion and a 109 bp tandem duplication. The spring growth habit is induced only by this deletion, since the deleted sequence contains the binding sites for a repressor protein similar to TaGRP2 in wheat, which reduces the expression level of the wild allele *Vrn-D1* in the absence of exposure to low temperatures [17,62]. As was shown previously, deletions or mutations leading to the appearance of positively charged amino acids in the CCT domain are responsible for the recessive of the *ZCCT-1* and *ZCCT-2* alleles, which account for the spring growth habit of wheat and barley [9,31,63]. Nonfunctional alleles of these genes in *Ae. tauschii* were found in a population originating from Iran [30,61]. These accessions contained an allelic variant of the *ZCCT*-*D1* gene with a 24 bp deletion in the first intron, located 6 bp upstream of the splicing acceptor site. Such a mutation alters splicing, resulting in the absence of translation of 35 amino acids in front of the CCT domain or in the translation of part of the intron [30]. For the dominant gene *ZCCT*-*D2*, a shift of the reading frame was shown, which was due to a 1 bp deletion in the second exon, and for that reason the CCT domain in the translated protein was not observed [30]. These mutations in the structure of the *ZCCT*-*D1* and *ZCCT*-*D2* genes cause dysfunction of the proteins they encode.

No mutant alleles of the *Ppd* genes with a confirmed effect on photoperiod sensitivity have been detected in *Ae. tauschii* [37,64,65,66]. In common wheat, the dominant allele *Ppd-D1a* with a 2089 bp deletion in the promotor region has been described in the D genome, due to which plants were able to complete ontogenesis under short-day conditions [37]; however, this allele appeared in the *T. aestivum* genome independently of *Ae. tauschii* [37,64,65,66]. There is only one report about the discovery of a dominant allele of the *Ppd-D1* gene in *Ae. tauschii* [67]; however, nothing is known about the molecular characteristics of this allele.

As research into the genetics of *Ae. tauschii* is limited compared to common wheat, the alleles of its genes that affect vegetative period duration remain unknown [26,61,62], although they could be introgressed into commercial cultivars. 

The aim of this work was to study the alleles of the *Vrn* and *Ppd* genes that affect the duration of the vegetative period in spring accessions of *Ae. tauschii*. As a result, two allelic variants of the *Vrn-D1* gene have been found that differ from their wild-type counterpart. One variant of this gene had a known deletion (5437 bp) in the first intron. The other allele had a previously undescribed 3273 bp deletion in the first intron. Using real-time PCR (qPCR), we showed significant differences in expression level between the new allelic variants of the *Vrn-D1* gene and the intact allele. 

## 2. Results

### 2.1. Growth Habit of the Ae. tauschii Accessions

The growth habit (spring/winter) was determined for 15 accessions of *Ae. tauschii*, eleven of which had spring habit, and four had winter habit (Table 1). The spring growth habit has been previously studied for four accessions: KU-2009 (Pakistan), K-608 (Georgia), K-992 (Afghanistan) and K-864 (origin unknown) [26], and the same was confirmed in the present study. In addition to these four, all other spring accessions developed spikes without preliminary vernalization no later than 20 days after the heading of the spring control accession, so they were classified as spring growth-habit accessions. The Palestinian, Chinese and Iranian accessions and the winter control K-1740 did not develop spikes 80 days after the heading of the spring accession KU-20-6.

The significance of differences in vegetative period duration was confirmed using the LSD_0.05_ criterion calculated with a one-way analysis of variance (ANOVA). It was found that accessions K-608, C21-51-43, C21-5118, C21-4030, C21-5112 were significantly superior to the spring control KU-20-6 in days from planting to heading (Table 1). No significant differences in the timing of heading were found between the winter accessions and the winter control K-1740 (Table 1). 

### 2.2. Determination of the Structure of the Vrn Genes

**Figure 2:**
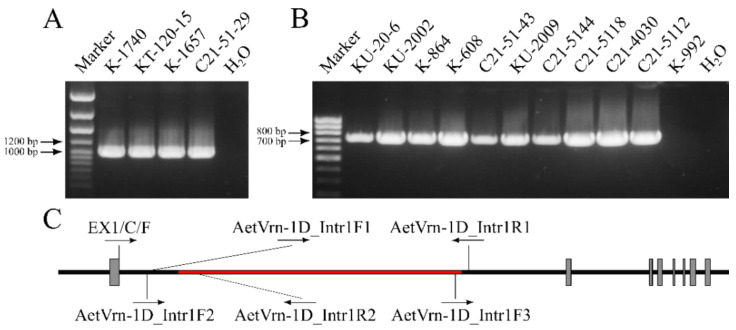
(**A**): Electrophoresis of the PCR products of the first intron of the *Vrn-D1* gene with the primers AetVrn-D1_Intr1F2 and AetVrn-D1_Intr1R2. The 998 bp long region indicates the presence of an intact intron. (**B**): Electrophoresis of the PCR products of the first intron of the *Vrn-D1* gene with the primers AetVrn-D1_Intr1F1 and AetVrn-D1_Intr1R1. The 747 bp long region indicates the presence of a 5437 bp deletion in the first intron. (**C**): The scheme of the *Vrn-D1* gene and the position of the primers used. Gray rectangles, exons; black rectangles, introns; the 5437 bp deletion is in red.

In each of all 15 *Ae. tauschii* accessions studied, the structure of the first intron of the *Vrn-D1* gene was analyzed. To determine the presence of an intact intron in the winter accessions, the PCR analysis was performed using the primers AetVrn-D1_Intr1F2 and AetVrn-D1_Intr1R2 (Figure 2C). For each winter accession, a 998 bp PCR product was obtained, indicating the presence of an intact intron (Figure 2A). In the spring accessions, the PCR analysis was performed to determine the presence of a 5437 bp deletion in the first intron (with the primers AetVrn-D1_Intr1F1 and AetVrn-D1_Intr1R1) (Figure 2C). For all accessions except the K-992 from Afghanistan, a 747 bp PCR product similar to that for the control KU-20-6 was obtained, which indicates the presence of this deletion in the first intron of these accessions (Figure 2B). No PCR product was found in K-992 (Figure 2B). 

It has been previously discovered that the spring growth habit can be formed due to small deletions in the region of the first intron of the *ZCCT-D1* gene and the region of the second exon of the *ZCCT-D2* gene. Therefore, we examined these critical gene regions in our accessions [30]. PCR with the primer pairs AetZCCT-D1_Intr1F1, AetZCCT-D1_Intr1R1, and AetZCCT-D2_Ex2F1, AetZCCT-D2_Ex2R1 primers gave 302 bp and 539 bp products, respectively. Sequencing of the PCR products and alignment of the *ZCCT-D1* and *ZCCT-D2* sequences presented in GenBank showed the absence of any other mutation in these regions (information on the sequences obtained is given in the Data Availability Statement section). 

### 2.3. Determination of the Structure of the Ppd Genes

**Figure 3:**
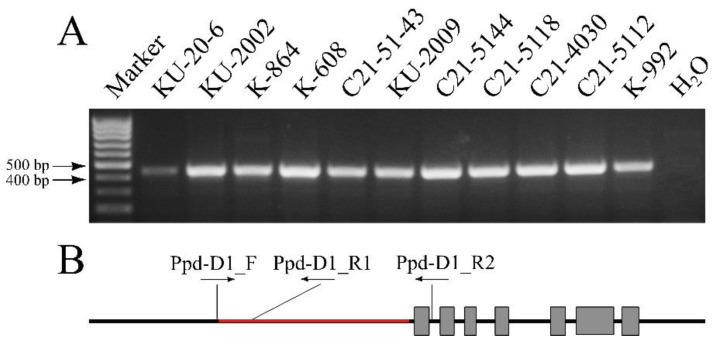
(**A**): Electrophoresis of PCR products of the promoter region of the *Ppd-D1* gene with the primers Ppd-D1_F and Ppd-D1_R1. The presence of a 454 bp fragment indicates the absence of deletions upstream of the *Ppd-D1* gene promoter. (**B**): Scheme of the *Ppd-D1* gene and the position of the primers used. Gray rectangles, exons; black rectangles, introns; deletion 2089 bp in the promoter region is highlighted in red.

In the spring accessions, the PCR analysis was performed with the primers Ppd-D1_F and Ppd-D1_R2 to determine the presence of a 2089 bp long deletion in front of the coding region of the *Ppd-D1* gene. No PCR product was found in any of the accessions. PCR amplification using the primers Ppd-D1_F and Ppd-D1_R1 gave a 454 bp product, which indicates the presence of an intact region of this gene (Figure 3).

### 2.4. Analysis of the Nucleotide Sequence of the Intron of the Vrn-D1 Gene

Because the previously described mutations in the *Vrn*-*D1*, *ZCCT-D1* and *ZCCT-D2* genes were not found in spring accession K-992 and also because mutations in the *VRN-1* genes are most often found in spring-type cereals [8,11,18,19,20,21,22,23,24,25,26], we explored the structure of the *Vrn-D1* gene in K-992. Mutations responsible for the spring growth habit are localized in the promoter region or in the first intron of the *VRN-1* genes. It was previously shown that the promoter region of the *Vrn-D1* gene in K-992 does not contain any disorders [26]. For that reason, we carried out a PCR analysis with the primers AetVrn-D1_Intr1F2 and AetVrn-D1_Intr1R2 (Figure 2C) to confirm the presence of an intact allele of *vrn-D1*, but no PCR product was found. Presumably, the first intron of the *Vrn-D1* gene in K-992 had a different deletion, which was shorter than the 5437 bp deletion in KU-20-6, but covered the binding region for the repressor protein identified in the spring varieties of wheat and barley [11,68,69]. We developed a direct primer AetVrn-D1_Intr1F3 lying outside the critical region while being within the KU-20-6 deletion (Figure 2C). PCR analysis with the primers AetVrn-D1_Intr1F3 and AetVrn-D1_Intr1R1 gave a 275 bp product, which indicated the presence of a previously undescribed deletion in the first intron of the *Vrn-D1* gene in accession K-992.

**Figure 4:**
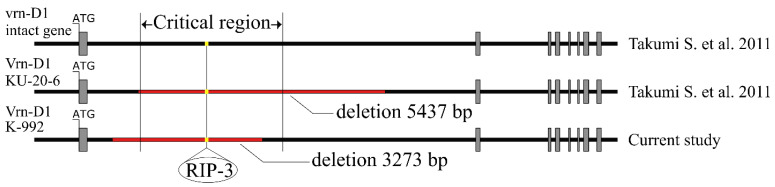
Position of deletions of the first intron of two allelic variants of the *Vrn-D1* gene in *Ae. tauschii.* Gray rectangles, exons; black bars, introns; red bars, deletions. Yellow dots indicate the sequence of the RIP-3 region in the first intron. The designated link "Takumi S. et al. 2011" is located in the "References" section under number [62]. The critical region is the region of the first intron, where deletions lead to the development of the spring growth habit in wheat and barley [11,68,69].

To determine the exact location of the deletion, we performed PCR amplification with the direct primer Ex1/C/F and the reverse primer AetVrn-D1_Intr1R1 (Figure 2C). The resulting 3500 bp PCR product was sequenced by the Sanger method using a forward and a reverse primer. The nucleotide sequence determined using the primer AetVrn-D1_Intr1R1 did not differ from its matching region in the *Vrn-D1* gene, so we came to a conclusion that the deletion is located at the 5’ end of the first intron. Sequencing using the primer Ex1/C/F and alignment against the nucleotide sequence of the intact allele of the *Vrn-D1* gene in *Ae. tauschi* showed that K-992 contained a 3273 bp deletion in the first intron of the *Vrn-D1* gene, which does not fully include the critical region that is responsible for the spring growth habit in wheat and barley [11,68,69] (Figure 4). It was also found that this deletion includes the RIP-3 sequence, an important region of the gene, affecting the degree of binding of the pre-mRNA gene to the protein TaGRP2 [13] (Figure 4). 

The results of the study of the first intron structure of the *Vrn-D1* gene, the study of the structure of the promoter region of the *Ppd-D1* gene, and the determination of the nucleotide sequences of the first intron of the *ZCCT-D1* gene and the second exon of the *ZCCT-D2* gene are presented in Table 2.

### 2.5. Study of the Relative Expression Levels of Different Variants of the Vrn-D1 Gene in Ae. tauschii

To study the effect of the 3273 bp deletion on the expression level of the *Vrn-D1* gene and to compare the expression profiles of different variants of this gene, we grew plants of three accessions: (i) winter K-1740, (ii) spring KU-2009 and (iii) spring K-992.

**Figure 5:**
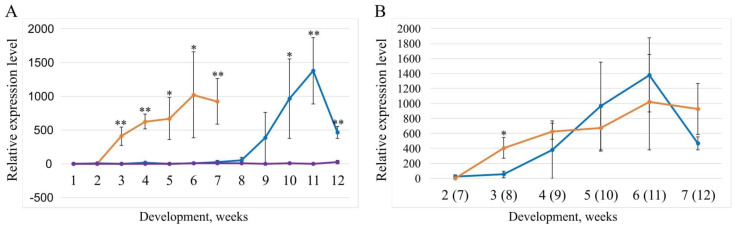
Change in the relative expression level of accessions with different alleles. Violet color indicates accession K−1740, blue—KU−2009, orange—K−992. Data for all accessions are expressed as mean value ± standard deviation (n = 3). Student’s *t*−test was used for testing of statistical significance (* *p* < 0.05, ** *p* < 0.01). (**A**): comparison of K−1740, KU−2009 and K−992. (**B**): comparison of K−992 and KU−2009 five weeks before heading. The expression profile is shown from the second week of development for K−992 and from the seventh week for KU−2009.

Days from planting to heading in the spring accessions KU-2009 and K-992 was 81 ± 9.4 and 48.5 ± 1.8 (t = 10.7), respectively, so we collected leaves for RNA isolation only until week 12 for KU-2009 and till week 7 for K-992. Winter plants K-1740 did not develop spikes during the entire vegetative period, so in this case we collected leaves for RNA isolation only till week 12, as we did for KU-2009. A quantitative comparison of the expression profiles revealed differences in the transcription levels of the *Vrn-D1* gene in the leaves of different accessions at different vegetative periods (Figure 5). In K-1740, the *Vrn-D1* expression level was low during all 12 weeks of development. Significant differences in *Vrn-D1* expression level were revealed between the spring accession KU-2009 and the winter accession on weeks 10, 11 and 12 of development (Figure 5A); between the spring accession K-992 and each of K-1740 and KU-2009, starting from week 3 of development and throughout all subsequent weeks (Figure 5A). A comparison of *Vrn-D1* expression level between K-992 and KU-2009 on week 5 before heading revealed a significantly higher number of transcripts in K-992 on week 4 before heading (Figure 5B). In the later weeks before heading, the differences in expression levels between different alleles were not significant (Figure 5B). 

## 3. Discussion

Mutations in the *Vrn* and *Ppd* genes are of great importance for early heading [3,4,45], so the study of their various allelic variants is very important for the breeding of early maturing cultivars. *Ae. tauschii* populations have spring accessions, in which mutant *Vrn* genes appeared independently of those in *T. aestivum* [30,61,62]. Such alleles are very important for the transfer into common wheat, as they reduce the vegetative period of plants [30]. For this reason, our primary plan was to determine which growth habit *Ae. tauschii* has in order to be able to study the allelic composition of the *Vrn* and *Ppd* genes in the spring accessions. 

In 11 spring accessions, the structure of the first intron of the *Vrn-D1* gene was studied. Ten of them had a 5437 bp deletion in the first intron, which had already been detected in *Ae. tauschii* [62]. It has been shown that this deletion accounts for a spring growth habit, which is consistent with the studies carried out on common wheat [11,68,69] and was additionally confirmed by our results (Figure 2, Table 1 and Table 2). None of the accessions that we have studied had other mutations in the *ZCCT*-*D1*, *ZCCT*-*D2* or *Ppd-D1* genes. One spring accession, K-992, had an allele of the *Vrn-D1* gene that differed from that described in KU-20-6. As revealed by sequencing, K-992 had a 3273 bp deletion in the first intron of the *Vrn-D1* gene, which partially covered the binding region for the repressor protein identified in some spring varieties of wheat and barley [11,68,69]; this deletion also included the RIP-3 region, nucleotide substitutions that had a great impact on the degree of binding of the protein TaGRP2 to the pre-mRNA of the wheat *VRN-1* genes [13]. Although this deletion does not completely cover the critical region of the first intron, it can be assumed that due to the removal of the RIP-3 region from the sequence, this deletion can influence the expression of the *Vrn-D1* gene in the leaves. To test this assumption, we measured the relative expression level of the *Vrn-D1* gene. 

When grown in field conditions, the winter accession used as the control did not ear as it possessed the recessive allele *vrn-D1* with a low basal level of expression. Plants of spring accessions K-992 and KU-2009 with deletions in the first intron of the *Vrn-D1* gene eared without prior exposure to vernalization and had a significantly higher amount of mRNA of the *Vrn-D1* gene in the leaves than the winter control had. These results support the assumption that the 3273 bp deletion in the first intron of the *Vrn-D1* gene causes expression without having to expose the plants to low temperatures, leading *Ae. tauschii* to develop a spring growth habit. The nucleotide sequences of both deletions in the first intron of the *Vrn-D1* gene identified in spring accessions K-992 and KU-2009 as well as the sequences of the deletions in the first intron of the *VRN-1* genes found in the spring wheat and barley plants contain the RIP-3 region [11,68,69]. It was previously shown that nucleotide substitutions in this region can cause a weaker binding of the protein TaGRP2 to the nucleotide sequence [13], which results in a weaker plant response to vernalization [70]. The *Vrn-D4* gene located in the D genome of common wheat is a copy of the *Vrn-A1* gene [12]. It has an allelic variant with three nucleotide substitutions in the RIP-3 region, which forms a very weak bond with TaGRP2 and determines the expression of this gene even though there are no deletions in the first intron [13]. Based on the available data, it can be assumed that the RIP-3 region is a key sequence required for binding the GRP2 family repressor protein to the pre-mRNA genes of the *VRN-1* locus in cereal plants. Our data indirectly agree with the finding, since the sequence of the 3273 bp deletion found in the first intron of the *Vrn-D1* gene in spring accession K-992 includes the RIP-3 region but does not completely cover the previously identified deletions conferring a spring growth habit on cereals [11,68,69]. 

When grown in field conditions, K-992 plants formed inflorescences earlier than KU-2009 plants, while the expression level of the *Vrn-D1* gene in K-992 became significantly different from that of the KU-2009 allele as early as starting from week 3 of development. Such a relationship between the time of onset of *VRN-1* expression and the heading time has been shown previously [20] and now was additionally demonstrated in our study. The difference in the time of heading and the time of appearance of the first transcripts of the *Vrn-D1* gene between K-992 and KU-2009 is unquestionable; however, this is most likely not due to the difference in the allelic variants of the *Vrn-D1* gene.

It may be noted that deletions in the first intron of the *Vrn-D1* gene in all spring accessions analyzed in this study were detected, but there was not a single spring accession containing other known alleles of the *Vrn* and *Ppd-D1* genes that differed from the wild types (Table 2). At the same time, several spring accessions with an allelic variant of the *Vrn-D1* gene identical to that in KU-20-6 were significantly superior to the spring control in vegetative period duration (Table 1). The difference in vegetative period duration observed among the spring accessions of *Ae. tauschii* with the same 5437 bp deletion in the first intron of the *Vrn-D1* gene has been described previously [62] and was confirmed in our study. Consequently, the presence of the same allelic variant of the *Vrn-D1* gene in different spring accessions of *Ae. tauschii* did not form the same length of the growing season.

Spring accessions K-992 and KU-20-6 have *Vrn-D1* alleles but do not differ significantly from each other in the duration of the vegetative period (Table 1 and Table 2). Monogenic control of the growth habit in K-992 has been shown previously [71], which completely excludes the presence of other *Vrn* alleles in it, except for the allele of the *Vrn-D1* gene that we discovered. Thus, taking into account all the above evidence, it can be assumed that the plants of the accessions we studied have different undescribed allelic variants of the genes that have an impact on vegetative period duration rather than growth habit. However, as entries in Table 1 confirm, the 3273 bp deletion in the first intron of *Vrn-D1* accounts for the development of a spring growth habit by the *Ae. tauschii* accession. The effect of this deletion on the expression time of the *Vrn-D1* gene and the earliness of plants can only be speculated and still remains to be confirmed.

## 4. Materials and Methods

### 4.1. Plant Material, Growth Conditions, Assessment of Vernalization Requirements and Determination of Heading Time

A total of 15 accessions of *Ae. tauschii* from various regions were taken as the material for the study. The seed progeny was obtained from each accession to assess the growth habit and heading time. A total of 10 seeds were sown per accession at the hydroponic greenhouse of the Institute of Cytology and Genetics of the Siberian Branch of the Russian Academy of Sciences without preliminary vernalization. Plants were grown at a temperature of 23–25 °C and a long day (16 h). KU-20-6 with the dominant allele of the *Vrn-D1* gene was used as the spring control (plants that head without vernalization) [62]. K-1740 with the intact allele of the *Vrn-D1* gene was used as the winter control (plants that do not head without vernalization). Plants that did not ear without vernalization were transferred to a cold chamber and grown at 3–4 °C and 16 h photoperiod for 50 days, after which the growing conditions were set to standard (23–25 °C and 16 h photoperiod) and the plants were observed to be heading (such plants were referred to as winter plants). 

The number of days from planting to heading was recorded for each plant individually. Based on the data obtained, the average value for each accession was calculated. The significance of the differences in vegetative period duration was confirmed by the difference in the sample means between the accessions and controls for each group (spring type/winter type) compared to the smallest significant difference (LSD_0.05_) calculated by one-way analysis of variance. Accession voucher, place of collection, growth habit and heading time for each accession are presented in Table 1.

### 4.2. Isolation of Total DNA, PCR Amplification and Determination of Nucleotide Sequences of the Vrn-D1, ZCCT-D1, ZCCT-D2 and Ppd-D1 Genes

Isolation of total DNA was carried out using the DNeasy Plant Mini Kit (QIAGEN, Hilden, Germany) according to the manufacturer’s protocol. For extraction of DNA, 50–100 mg of leaves taken from plants of each accession were used. The quantity and quality of isolated DNA were determined using the NanoDrop2000 (Thermo Scientific, Waltham, MA, USA) spectrophotometer and electrophoretic separation in 1% agarose gel containing ethidium bromide (0.5 mg/mL) in 1 × TAE. The primer pairs used for PCR amplification and annealing temperatures are provided in Supplementary Materials Table S1.

Polymerase chain reactions (PCR) were carried out in a volume of 20 µL with BioMaster LR HS-PCR-Color (2×) (Biolabmix, Novosibirsk, Russia), 10 pmol of each primer, and 30 ng of genomic DNA. Separation of amplification products was carried out in 1% agarose gel. PCR fragments obtained with the primer pairs AetZCCT-D1_Intr1F1, AetZCCT-D1_Intr1R1; AetZCCT-D2_Ex2F1, AetZCCT-D2_Ex2R1 and Ex1/C/F, AetVrn-D1_Intr1R1 were isolated from the gel using the QIAquick Gel Extraction Kit (QIAGEN, Germany) and then sequenced. Sequencing was completed using 200 ng of the product and the BigDye Terminator v3.1 Cycle Sequencing Kit (Thermo Scientific, USA) on the ABI 3130XL genetic analyzer (Applied Biosystems, Foster City, CA, USA) at the SB RAS Center for Genomics (URL: http://www.niboch.nsc.ru/doku.php/corefacility (accessed on 15 May 2023)).

### 4.3. Analysis of the Expression Level of the Vrn-D1 Gene, Isolation of Total RNA, cDNA Synthesis, Real-Time PCR Amplification and Evaluation of the Expression Level

To analyze the expression level at the experimental field of the Institute of Cytology and Genetics of the Siberian Branch of the Russian Academy of Sciences, plants of three accessions were grown: spring accession K-992, spring accession KU-2009 and winter accession K-1740. Ten seeds of each accession were sown and not exposed to low positive temperatures (they were not vernalized). The number of days from sowing to heading in the spring plants was recorded for each plant individually, after which the mean sample value was calculated for each spring accession and the significance of differences was assessed using the Student’s *t*-test. Winter plants were used as the control with a low basal value of the *Vrn-D1* expression. Each week of plant growth from planting to heading, a leaf of the main shoot was picked. Since several leaves were formed on the shoots of *Ae. tauschii*, it was possible to obtain fresh material for RNA isolation throughout the entire vegetative period. The collection was made in the middle of the day to avoid the influence of circadian rhythms. Leaves of all accessions were frozen in liquid nitrogen and stored at −80 °C until use.

Total RNA was isolated from leaves using the LRU-100-50 kit (Biolabmix, Russia) according to the manufacturer’s instructions. Leaves of three plants taken from each accession (three biological replicates) once per week were taken for 12 weeks. The concentration of isolated RNA was measured using the NanoDrop2000 (Thermo Scientific, USA) spectrophotometer and the quality of RNA was checked by electrophoretic separation in 1% agarose gel. cDNA was synthesized using the RNAscribe RT kit (Biolabmix, Russia), according to the manufacturer’s instructions, after which it was diluted 5 times and used as a template for real-time PCR.

Real-time PCR was performed in the CFX96 Real-Time Touch amplifier (Bio-Rad, Hercules, CA, USA) with SYBR Green (Biolabmix, Russia) in three technical replicates. To study the relative expression level of the *Vrn-D1* gene, we used three endogenous control genes: *Actin* (*ACTIN*), *Glyceraldehyde-3-phosphate dehydrogenase* (*GAPDH*), and *S-adenosylmethioninedecarboxylaseproenzyme* (*SAMD*). The primers for the *ACTIN* and *SAMD* genes were as in previous studies [25,72]. For amplification of the *GAPDH* gene region, we developed original primers. For amplification of the *Vrn-D1* gene, we also designed new primers that amplified the region of the seventh and eighth exons of the gene, similar to the pair of primers designed to study the expression of the *Vrn-D1* gene in common wheat [73]. The sequences of the primer pairs used for real-time PCR are presented in additional file 1: Table S2. The annealing temperature was the same for all primer pairs. Primer efficiency was tested by a series of dilutions of the cDNA template. A melt-curve protocol was performed for each primer pair to detect specific products. The expression level was calculated relative to the geometric mean of the expression levels of three endogenous control genes (*ACTIN*, *GAPDH*, *SAMD*) [74]. The values obtained were used to plot the change in the relative expression level of the *Vrn-D1* gene in three accessions. The significance of the differences in expression level was confirmed using the Student’s *t*-test.

## 5. Conclusions

In this work, we explored the allelic composition of the *Vrn* and *Ppd* genes in 15 accessions of *Ae. tauschii*. Mutant alleles were found only for the *Vrn-D1* gene, including a previously undescribed allelic variant with a 3273 bp deletion in the first intron. We found that the expression level of the new allele was significantly higher than that of the wild-type gene when the plants were grown without vernalization. Furthermore, the location of the detected deletion narrows the size of the DNA sequence in the first intron, in which the deletions lead to a spring growth habit in *Ae. tauschii.* The new allele can be introgressed into common wheat to enhance the biodiversity of a spring growth habit and vegetative period duration of wheat cultivars.

## Figures and Tables

**Table 1:** *Ae. tauschii* accessions used in the study, their growth habit (S, spring; W, winter), vegetative period and the significance of differences.

Accession Number	Accession Location	Growth Habit	Days to Heading, Mean ± Standard Deviation	d ≥ LSD_0.05_
KU-20-6 (Control)	Pakistan	S	65 ± 5.2	
KU-2002	Pakistan	S	67.5 ± 4.7	
K-864	unknown	S	73.1 ± 2	
K-608	Georgia	S	81.4 ± 7.6	*
C21-51-43	Pakistan	S	84.8 ± 11	*
KU-2009	Pakistan	S	72.8 ± 7.6	
C21-5144	Pakistan	S	70.8 ± 5.8	
C21-5118	unknown	S	78.8 ± 3.7	*
C21-4030	unknown	S	77.1 ± 5.95	*
C21-5112	unknown	S	82 ± 9.9	*
K-992	Afghanistan	S	57 ± 5.9	
LSD_0.05_			9.87	
K-1740 (Control)	Afghanistan	W	224.8 ± 4.1	
KT-120-15	China	W	211.5 ± 7.2	
K-1657	Palestine	W	221.3 ± 5.3	
C21-51-29	Iran	W	226.7 ± 7.1	
LSD_0.05_			6.73	

* Significant differences between accessions and controls are indicated.

**Table 2:** Structural changes of the genes that control the duration of the vegetative period of the *Ae. tauschii* accessions studied.

Accession Number	*Vrn-D1* First Intron	*ZCCT-D1* First Intron	*ZCCT-D2* Second Exon	*Ppd-D1* Promoter
KU-20-6	5437 bp deletion	intact	intact	intact
KU-2002	5437 bp deletion	intact	intact	intact
K-864	5437 bp deletion	intact	intact	intact
K-608	5437 bp deletion	intact	intact	intact
C21-51-43	5437 bp deletion	intact	intact	intact
KU-2009	5437 bp deletion	intact	intact	intact
C21-5144	5437 bp deletion	intact	intact	intact
C21-5118	5437 bp deletion	intact	intact	intact
C21-4030	5437 bp deletion	intact	intact	intact
C21-5112	5437 bp deletion	intact	intact	intact
K-992	3273 bp deletion	intact	intact	intact
K-1740	intact	nd	nd	nd
KT-120-15	intact	nd	nd	nd
K-1657	intact	nd	nd	nd
C21-51-29	intact	nd	nd	nd

## Data Availability

The *ZCCT-D1* gene sequences were deposited to the NCBI GenBank database under accession numbers: OR160365 (C21-51-43), OR160366 (C21-4030), OR160367 (C21-5112), OR160368 (C21-5144), OR160369 (KU-20-6), OR160370 (K-608), OR160371 (K-864), OR160372 (K-992), OR160373 (KU-2002), OR160374 (KU-2009), OR160375 (C21-5118). The *ZCCT-D2* gene sequences were deposited to the NCBI GenBank database under accession numbers: OR160376 (C21-51-43), OR160377 (C21-4030), OR160378 (C21-5112), OR160379 (C21-5144), OR160380 (KU-20-6), OR160381 (K-608), OR160382 (K-864), OR160383 (K-992), OR160384 (KU-2002), OR160385 (KU-2009), OR160386 (C21-5118). The sequence of the first intron of the *Vrn-D1* gene with a 3273 bp deletion found in the K-992 was deposited to the NCBI GenBank database under accession number OR160387.

## Supplementary Materials

The following supporting information can be downloaded at: https://www.mdpi.com/article/10.3390/plants12203596/s1, Table S1. Primers were used to study the structure of the *Vrn-D1, ZCCT-D1*, *ZCCT-D2* and *Ppd-D1* genes. Table S2. Primers were used to analyze the expression level of the *Vrn-D1* gene.
