# Supplementary material for: Analysis of the Structural Organization and Expression of the Vrn-D1 Gene Controlling Growth Habit (Spring vs. Winter) in Aegilops tauschii Coss
Source: Plants (Basel). 2023 Oct 17;12(20):3596. doi: 10.3390/plants12203596 (PMC10610194; doi:10.3390/plants12203596)
Supplement: Supplementary file 1 [file plants-12-03596-s001.zip › plants-2611271-supplementary.pdf]

Table S1. Primers used to study the structure of the *Vrn-D1*, *ZCCT-D1*, *ZCCT-D2* and *Ppd-D1* genes.

Primer name	Primers sequence	Target sequence	Annealing Temp. °C	Product Size (bp)	Reference
AetVrn-D1_Intr1F1<br>AetVrn-D1_Intr1R1	TGTCTGCCTCATCAAATCCGTG<br>AGGATTGAAGCGAGAGCAGAGG	*Vrn-D1* intron 1<br>with 5437 bp deletion	62	747	[62]
AetVrn-D1_Intr1F2<br>AetVrn-D1_Intr1R2	GTTGTCTGCCTCATCAAATCC<br>AAATGAAAAGGAACGAGAGCG	*Vrn-D1* intron 1<br>without deletion	61	998	[62]
AetVrn-D1_Intr1F3<br>AetVrn-D1_Intr1R1	ACAGACCACCAGTGACCGAC<br>AGGATTGAAGCGAGAGCAGAGG	*Vrn-D1* intron 1<br>for new deletion	62	275	Current study<br>[62]
Ex1/C/F<br>AetVrn-D1_Intr1R1	GTTCTCCACCGAGTCATGGT<br>AGGATTGAAGCGAGAGCAGAGG	*Vrn-D1* intron 1<br>for new deletion	62	3500	[11]<br>[62]
AetZCCT-D1_Intr1F1<br>AetZCCT-D1_Intr1R1	AGTTGTTATCTTCCGCTGTC<br>CTCTCCTGCATTGTGGGATA	*ZCCT-D1* intron 1<br>with 24 bp deletion	61	302 or<br>278	Current study
AetZCCT-D2_Ex2F1<br>AetZCCT-D2_Ex2R1	ACTGTGTGCTTCTTTGGACAC<br>TACCGGAACCATCCGAGG	ZCCT-D2 exon 2<br>with 1 bp deletion	63	539 or<br>538	Current study
Ppd-D1_F<br>Ppd-D1_R1<br>Ppd-D1_R2	ACGCCTCCCACTACACTG<br>GTTGGTTCAAACAGAGAGC<br>CACTGGTGGTAGCTGAGATT	*Ppd-D1* promoter region<br>with 2089 bp deletion	54	454 or<br>228	[37]

Table S2. Primers used to analyze the expression level of the *Vrn-D1* gene.

Primer name	Primers sequence	Primer efficiency	Annealing Temp. °C	Reference
qAetVrn-D1_F4<br>qAetVrn-D1_R4	GCTCTTCTTCTTCTTCCTTCAT<br>GGCTCACCATCCACAGT	90,93%	63	Current study
qAetGAPDH_F1<br>qAetGAPDH_R1	TTCGGAATCGTGAAGGGGAC<br>CTCGTTGGCACGATGTTCAG	90,67%	63	Current study
qSAMDC_F<br>qSAMDC_R	TACTTCACAGCCGATAGC<br>CCGATTGTTGGACGATACT	93,44%	63	[72]
qACTIN_F<br>qACTIN_R	ATGGAAGCTGCTGGAATCCAT<br>CCTTGCTCATACGGTCAGCAATAC	107,4%	63	[25]
